# Elevated Mutation Rate during Meiosis in *Saccharomyces cerevisiae*

**DOI:** 10.1371/journal.pgen.1004910

**Published:** 2015-01-08

**Authors:** Alison Rattray, Gustavo Santoyo, Brenda Shafer, Jeffrey N. Strathern

**Affiliations:** Gene Regulation and Chromosome Biology Laboratory, NCI-Frederick, FNLCR, Frederick, Maryland, United States of America; National Cancer Institute, United States of America

## Abstract

Mutations accumulate during all stages of growth, but only germ line mutations contribute to evolution. While meiosis contributes to evolution by reassortment of parental alleles, we show here that the process itself is inherently mutagenic. We have previously shown that the DNA synthesis associated with repair of a double-strand break is about 1000-fold less accurate than S-phase synthesis. Since the process of meiosis involves many programmed DSBs, we reasoned that this repair might also be mutagenic. Indeed, in the early 1960′s Magni and Von Borstel observed elevated reversion of recessive alleles during meiosis, and found that the revertants were more likely to be associated with a crossover than non-revertants, a process that they called “the meiotic effect.” Here we use a forward mutation reporter (*CAN1 HIS3*) placed at either a meiotic recombination coldspot or hotspot near the *MAT* locus on Chromosome III. We find that the increased mutation rate at *CAN1* (6 to 21 –fold) correlates with the underlying recombination rate at the locus. Importantly, we show that the elevated mutation rate is fully dependent upon Spo11, the protein that introduces the meiosis specific DSBs. To examine associated recombination we selected for random spores with or without a mutation in *CAN1*. We find that the mutations isolated this way show an increased association with recombination (crossovers, loss of crossover interference and/or increased gene conversion tracts). Polζ appears to contribute about half of the mutations induced during meiosis, but is not the only source of mutations for the meiotic effect. We see no difference in either the spectrum or distribution of mutations between mitosis and meiosis. The correlation of hotspots with elevated mutagenesis provides a mechanism for organisms to control evolution rates in a gene specific manner.

## Author Summary

Meiosis, the cellular division that gives rise to germ cells, contributes to evolution by reassortment of parental alleles. This process involves recombination initiated by Spo11-induced double-strand breaks early in meiosis. The result is that germ cells from a single meiosis are different from either parent. Here we show that the DNA repair associated with meiotic recombination is inherently mutagenic, providing an additional source of variation that can contribute to evolution. This elevated mutagenesis requires the Spo11 protein, and the rate of mutagenesis correlates positively with the frequency of meiotic double-strand breaks. Furthermore, the mutations that arise show an increased level of associated crossovers, consistent with having been introduced during recombination. We speculate that there is an evolutionary drive to position essential genes in meiotic recombination coldspots for slow evolution, and genes that can afford to evolve more rapidly are placed near meiotic recombination hotspots.

## Introduction

Mutation is an important component of evolution. Organisms need to forge a fine balance between maintaining stasis while allowing enough flexibility so that some members of the population can survive when environmental change occurs. Mitotic DNA replication and repair is a highly accurate process with mutations arising only 3.8 or 6.4×10^−10^ per base pair per cell division for *URA3* and *CAN1* respectively [1], despite a large burden of continual endogenous and exogenous DNA damage (estimated to occur at a rate of 10^3^ to 10^6^ lesions per cell per day for most organisms [2]). Although mitotic mutations can result in reduced fitness and disease, such as cancer, it is the germ line mutations that contribute to the fitness of future generations and ultimately successful evolution. Our focus here is to determine the rate at which mutations arise as the cells traverse meiosis. An enigma exists between the fitness cost of having a sexual cycle and the near ubiquity of sex among eukaryotes. Asexual organisms are thought to be favored in the short term, but they eventually accumulate too many irreversible deleterious mutations for long-term survival (Muller's ratchet; [3]). It is hypothesized that sexual reproduction improves fitness over the long run via assortment, by providing increased genetic variability, and a mechanism by which deleterious mutations are masked or eliminated [4].

Meiosis differs from mitosis in that diploid cells undergo two consecutive cell divisions to produce germ cells. Meiosis is a highly choreographed process that involves homologous pairing and recombination resulting in the segregation of homologous chromosomes [5]. Recombination occurs during the first meiotic prophase. Meiosis II is similar to a mitotic division where sister chromatid centromeres are segregated from one another. Recombination is strongly induced in the first meiotic prophase by programmed DNA double-strand breaks (DSBs) that are introduced by the Spo11 type II topoisomerase [6]. In budding yeast, the number of DSBs is estimated to be ∼160 per cell [7] of which ∼35% result in crossovers [8], [9]. Meiotic recombination is not uniform across the genome, but rather occurs at either high or low levels, termed hotspots and coldspots respectively. The frequency of meiotic crossovers is positively correlated with the local frequency of Spo11-induced DSBs [10] that, in turn, appear to be influenced by the underlying chromatin context ([11], and references cited therein). Crossovers themselves are subject to crossover interference, where there are fewer than expected double crossovers near each other [12].

Our laboratory has previously demonstrated that repair of mitotic DSBs are accompanied by 100 to 1000-fold increase in mutations near the site of the break (Break Repair Induced Mutagenesis -BRIM) [13]–[15]. High levels of mutation have also been observed to occur during an HO induced mating type switching-like assay [16], break-induced replication (BIR) where mutations are found as much as 36 kb from the initiating break [17], or associated with fragile genomic sites [18]. Mutagenesis is also elevated during repair after telomere erosion [19], [20]. A review of mutagenesis associated with DSB repair can be found in [21]. Adaptive mutation is a phenomenon characterized by stress-induced increases in mutation rates (i.e. starvation), and is associated with increased recombination in both bacteria and yeast, and appears to function via a DSB repair pathway [22], [23].

The Rev3/Rev7 translesion DNA polymerase (Polζ) is important for the majority (50–75%) of spontaneous mutations in yeast [24]. We demonstrated that during repair of a mitotically introduced site-specific DSB, Polζ is important for >90% of all base substitution mutations, but only minimally important for the predominating frameshift mutations [13], [25]. The role of Rev3 in mutagenesis of other DSB induced assays is context dependent (see [21] for a review). In some assays mutagenesis is entirely dependent upon *REV3* [20], while in other assays it has an intermediate effect [17],[18],[25], or is not required [16]. It is not clear what causes Rev3 recruitment to only some DSBs, but one possibility is the length of ssDNA produced during repair. ssDNA is more susceptible to DNA damage than dsDNA, and synthesis on the damaged template may require a translesion polymerase [17], [20], [25]. Mutations in Pol ζ do not appear to affect sporulation or viability, although the Rev1 protein (found in complex with polζ [26]) has been shown to physically interact with Spo11 [27].

Since recombination during the first meiotic prophase proceeds via DSB repair, we wondered if meiotic recombination was also mutagenic. In the early 1960s Magni and Von Borstel [28] observed an increased level (6–20 fold) of reversion of auxotrophic alleles during yeast meiosis, a process they termed “the meiotic effect”. Subsequently, Magni [29] demonstrated that 71% of the revertants analyzed had an associated crossover while the expected crossover association was 15%. This suggested that the increased mutagenesis might be linked to meiotic recombination. Magni also used the *CAN1* gene as a forward mutation reporter [30]. The *CAN1* gene encodes the arginine permease and allows cells to take up the toxic arginine analog canavanine [31]. Thus, cells with a wild type allele of *CAN1* are sensitive to canavanine, whereas mutations that inactivate the permease render the cell resistant to canavanine. The experiments of Magni and von Borstel, while seminal, had three caveats that we address here. 1) For the reversion experiments the nature of the alleles used to score reversion is unknown, hence the required reversion events are also unknown. 2) For the experiments with *CAN1* the mutation rates in diploids cannot be measured. Based on Magni's previous results he assumed that the diploid mitotic mutation rate was the sum of each of the haploid mutation rates, an assertion that is unlikely because the two parents differed by ∼38 fold in their rates to canavanine resistance (from 1.4×10^−9^ to 5.3×10^−8^), suggesting that other factors influenced the measured mutation rate. Also we now know that recombination and repair pathways are different in a/α cells than in either a or α cells [32]–[34]. 3) Because the *CAN1* is located beyond any essential genes on the left arm of chromosome V, it opens a terminal deletion pathway for mutagenesis that may not be a general mechanism [35]. Several additional attempts have been made to confirm these observations but all suffered from similar caveats or insufficient data (see Discussion).

In the current study we have revisited the meiotic effect using a diploid with a single *CAN1* gene coupled to a *HIS3* gene so that by maintaining selection for His^+^ cells cannot become canavanine resistant simply by loss of heterozygosity (LOH). Importantly, we find that the meiotic effect is entirely dependent upon Spo11, consistent with the idea that mutations are introduced during DSB repair. The location where we place the *CAN1 HIS3* cassette affects the rate of mutation in a manner consistent with frequency of recombination at that locus. We speculate that organisms can control the rate of evolution of different genes by controlling their location relative to meiotic recombination hotspots.

## Results

### Experimental system for studying increased mutation during meiosis

**Figure 1:**
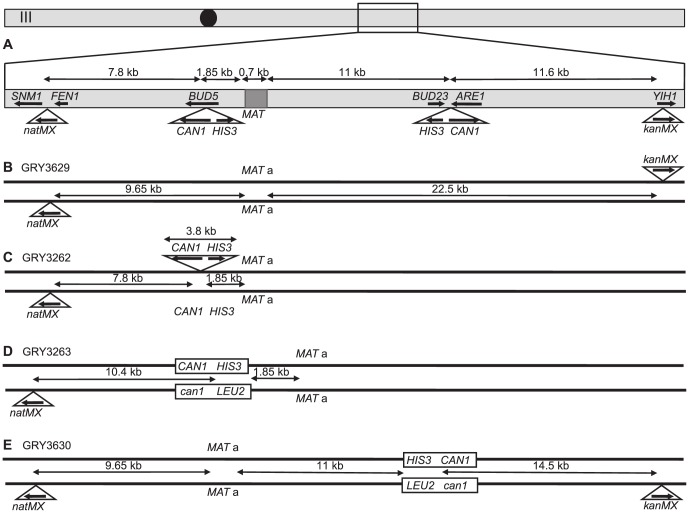
Schematic of strains used in this study. **A.** Map of chromosome III showing the two different locations that the cassettes were inserted into (independently), as well as the location of markers used for crossover analysis and the distances between them. **B.** Strain GRY3629 that only has the outside flanking markers to determine genetic intervals in the absence of the construct. **C.** Strain GRY3262 bearing a hemizygous insertion of the *HIS3 CAN1* cassette in the coldspot. **D.** GRY3263 bearing the substrate with homology to *can1* at the coldspot. **E.** GRY3630 bearing the substrate with homology to *can1* at the hotspot.

We constructed a 3.8 kb cassette containing wild type *HIS3* and *CAN1* such that the two genes are transcribed in opposite directions (Fig. 1). The normal *HIS3* and *CAN1* loci were deleted from the parental strains (see Materials and Methods for exact coordinates of each gene used). We inserted the 3.8 kb cassette into either of two different locations on chromosome III. Mutations were selected as His^+^ Can^r^ cells. For each experiment at least 18 colonies were grown to mid-log phase in rich media (see Materials and Methods for detailed experimental procedures). Initially, we measured the mutation rate in the haploids containing the substrate. We then made diploids carrying the *HIS3 CAN1* cassette. For the diploid strains, part of each culture was used to determine the mutation rates after mitotic growth while the remainder was transferred to sporulation medium for ∼5 days, and random spores (disrupted asci) were plated to determine the change in mutation rate after a single meiotic division. His^+^ Can^r^ mutants arising during mitotic growth were examined to determine if they were accompanied by crossovers as described in Materials and Methods, however we found very few crossovers among the mitotically arising events in the intervals being scored. His^+^ Can^r^ mutants existing in each culture prior to sporulation were subtracted from the total after meiosis to allow the measurement of mutations created during meiosis. This allows us to calculate a mutation rate per meiosis. Meiotic recombination was established by tetrad dissection. Because the mutation rate is low, we did not find any Can^r^ mutants in the tetrads we examined. By using random spores, we could plate many more cells. The starting strains were heterozygous for *ADE2/ade2 and CYH2/cyh2* (Table 1) allowing us to examine only red (*ade2-1*) cyh^r^ (*cyh2*) spores to help eliminate any cells that may have mated after plating. The overall frequency of red (Ade^-^) colonies was no different than the frequency of white (Ade^+^) colonies. The spores were then examined to determine location of crossovers as described in Materials and Methods.

**Table 1:** Yeast strains.

Strain	Genotype	Source
Haploids		
DC14	*MAT*a *his1*	CSH laboratory
DC17	*MAT*α *his1*	CSH laboratory
GRY633	*MAT*a *can1 his3-532*	This laboratory
GRY634	*MAT*α *can1 his3-532*	This laboratory
GRY1600	*MAT*a*-inc can1::hisG cry1 cyh2 his3*Δ*200 leu2*Δ*1 lys2::hisG trp1::hisG tyr7-1 ura3-52*	This laboratory
GRY1601	*MATα-inc can1::hisG cry1 cyh2 his3*Δ*200 leu2*Δ*1 lys2::hisG trp1::hisG tyr7-1 ura3-52*	This laboratory
GRY1673	*MAT*a*-inc can1::hisG cyh2 his3-*Δ*200 leu2-*Δ*1 lys*::*hisG trp1-*Δ*1 ura3-52 tyr 7-1*	This laboratory
GRY2690	*MAT*α *ade2*::*hphMX4 can1::hisG his3*Δ*200 leu2*Δ*1 trp*Δ*1 ura3Δ0 SMN1*::*natMX4*::*FEN1*	This study
GRY2691	GRY1673 *bud5*::*CAN1 HIS3*	This study
GRY3264	GRY2690 *bud5*::*pr-can1 LEU2*	This study
GRY3265	GRY2691 *rev3*::*LEU2*	This study
GRY3266	GRY2690 *rev3*::*LEU2*	This study
GRY3267	GRY2691 *spo13::kanMX*	This study
GRY3268	GRY2691 *spo11::kanMX*	This study
GRY3269	GRY2690 *spo11::kanMX*	This study
GRY3270	GRY2690 *spo13::hphMX*	This study
GRY3271	GRY2690 *spo11::kanMX spo13::hphMX*	This study
GRY3272	GRY2691 *spo11::kanMX spo13::hphMX*	This study
GRY3625	GRY1673 *BUD23::HIS3 CAN1::ARE1*	This study
GRY3626	GRY2690 *BUD23::LEU2 pr-can1::ARE1*	This study
GRY3627	GRY1673 *yih1::kanMX4*	This study
GRY3628	GRY3626 *yih1::kanMX4*	This study
GRY3631	GRY3625 *sae2::hphMX4*	This study
GRY3632	GRY3627 *sae2::hphMX4*	This study
GRY3633	GRY2690 *sae2::hphMX4*	This study
GRY3634	GRY3628 *sae2::hphMX4*	This study
GRY3863	GRY2691 *sae2::hphMX4*	This study
GRY3864	GRY3264 *sae2::hphMX4*	This study
Diploids		
GRY3262	GRY2690 X GRY2691	This study
GRY3263	GRY2691 X GRY3264	This study
GRY3273	GRY3268 X GRY3269	This study
GRY3274	GRY3267 X GRY3270	This study
GRY3275	GRY3271 X GRY3272	This study
GRY3276	GRY3265 X GRY3266	This study
GRY3629	GRY2690 X GRY3627	This study
GRY3630	GRY3625 X GRY3628	This study
GRY3635	GRY3633 X GRY3632	This study
GRY3636	GRY3631 X GRY3634	This study
GRY3865	GRY3863 X GRY3864	This study

### Mutation rate is elevated in meiosis

We first examined mutation rates in strains with the substrate inserted in the *BUD5* gene 1.85 kb centromere proximal to the *MAT*a locus on chromosome III (Fig. 1C; a schematic of the approximate location on Chromosome III is shown in Fig. 1A). The *MATα* strain harbors a proximal *natMX4* marker 7.8 kb proximal between the *SMN1* and *FEN1* genes.

We found that the mutation rate to Can^r^ was 2.8×10^−8^ in haploids (GRY2691 Table 2). Our mutation rate is somewhat (5.4-fold) lower than that calculated by Lang and Murray (1.5×10^−7^) [1]. However, Lang and Murray have also shown that the mutation level of the *URA3* gene can vary as much as six-fold dependent upon its location in the chromosome [36]. Thus, it is possible that the area where we are inserting the *CAN1 HIS3* cassette shows lower mitotic mutation rates than *CAN1* at its native locus. Also, it is possible that strain specific differences influence the mutation rates. Diploid cells had an ∼2 fold elevated mutation rate to 5.7×10^−8^ in an a/α diploid (GRY3262) during mitotic growth. In the diploid the cassette is hemizygous. Since both the haploid and the diploid have a single reporter at the same location, the difference between the mutation rates must be related to cell type and/or ploidy.

**Table 2:** Mutation rate at the *HIS3 CAN1* cassette in mitotic and meiotic cell divisions.

Strain (ploidy)^1^	Relevant genotype^2^	Location of *HIS3 CAN1* cassette	Homology to *CAN1*	Mitotic Rate^3,4^×10^8^	Meiotic Rate^3,5^×10^8^	Meiosis/Mitosis^6^
GRY2691 (H)	Wildtype	*BUD5*	NA^7^	2.8 (1.9–3.9)	NA	NA
GRY3262 (D)	Wildtype	*BUD5*	No	5.7 (4.3–7.2)	37 (29–69)	6.5
GRY3273 (D)	*spo11*	*BUD5*	No	3.1 (2.0–4.2)	NA	NA
GRY3274 (D)	*spo13*	*BUD5*	No	6.1 (4.0–8.2)	35 (28–65)	5.8
GRY3275 (D)	*spo11 spo13*	*BUD5*	No	6.5 (3.9–7.9)	7 (4.8–8.1)	1.1
GRY3265 (H)	*rev3*	*BUD5*	NA	0.8 (0.6–1.1)	NA	NA
GRY3276 (D)	*rev3*	*BUD5*	No	2.0 (1.2–2.3)	16 (12–19)	8.0
GRY3263 (D)	Wildtype	*BUD5*	Yes	8.2 (6.1–10.4)	49 (36–57)	5.9
GRY3625 (H)	Wildtype	*BUD23-ARE1*	NA	2.5 (2.0–4.1)	NA	NA
GRY3630 (D)	Wildtype	*BUD23-ARE1*	Yes	8.4 (6–11)	177 (110–260)	21.1

1 Ploidy H-haploid, D-diploid. ^2^Only relevant genotypes are shown, see Table 1 for full genotype. ^3^Values in parentheses are 95% confidence intervals (calculated as described in Materials and Methods). ^4^Mitotic rates calculated by MSS-MLE method [64], see Materials and Methods. ^5^Meiotic rates calculated as described in Materials and Methods. ^6^Meiosis/Mitosis is the calculated meiotic rate divided by the calculated mitotic rate to provide fold difference. ^7^NA- not applicable: haploids and *spo11/spo11* diploids are unable to sporulate.

To determine whether the insertion affected meiotic recombination, we compared meiotic crossovers in strains without and with the *HIS3 CAN1* cassette (Fig. 1B and 1C respectively; Table 3, GRY3269 and GRY3262). In the strain lacking the reporter cassette (GRY3269), recombination in the *natMX-MAT* interval was determined to be 5.6% or 2.8 cM, resulting in 0.29 cM/Kb. The recombination rate is lower than average for chromosome III (0.48 cM/Kb, http://www.yeastgenome.org/pgMaps/pgMap.shtml) confirming previous observations that this is a coldspot for recombination [37], [38]. Insertion of the *HIS3 CAN1* cassette further reduced recombination in the *natMX-MAT* interval to 3.2% or 1.6 cM, resulting in 0.16 cM/Kb (Table3, GRY3262). Note that the size of the heterologous insertion is not included in the length calculations for Table 3, since heterologous regions cannot participate in recombination. Any DNA break initiating within the heterology will either use a sister chromatid, or resect sufficiently to find homology thereby converting away the heterology [39]. The number of spores showing a crossover between MAT and *natMX* is not statistically significant between the two strains, (p = 0.17).

**Table 3:** Tetrad analysis near the *CAN1 HIS3* cassette.

Strain (location of *CAN1 HIS3*)	Interval	PD	NPD	TT	% recombination	cM^1^	Kb^2^	cM/Kb
GRY3629 No insert	*natMX* - *MAT*	269	0	16	5.6	2.8+/−0.6	9.7	0.29
	*MAT - kanMX*	140	1	144	51.2	26.3+/−1.8	22.5	1.2
GRY3262 *BUD5* ^3^	*natMX* - *MAT*	243	0	8	3.2	1.6+/−0.6	9.7	0.16
	*natMX – HIS3*	245	0	7	2.8	1.4+/−0.5	7.8	0.18
	*HIS3 - MAT*	252	0	3	1.2	0.59+/−0.3	1.85	0.32
GRY3276 *BUD5* ^3^	*natMX* - *MAT*	199	0	9	4.3	2.16+/−0.7	9.7	0.27
	*natMX – HIS3*	203	0	7	3.3	1.67+/−0.6	7.8	0.21
	*HIS3 - MAT*	211	0	1	0.5	0.24+/−0.24	1.85	0.13
GRY3263 *BUD5* ^4^	*natMX – MAT*	227	0	20	8.1	4.05+/−0.8	12.3	0.33
	*natMX – HIS3*	233	0	12	4.9	2.45+/−0.7	10.4	0.24
	*HIS3 - MAT*	240	0	6	2.4	1.22+/−0.4	1.85	0.66
GRY3630 *BUD23-ARE1* ^4^	*natMX* - *MAT*	296	0	14	9.7	2.26+/−0.6	4	0.23
	*MAT – HIS3*	246	0	61	19.8	9.93+/−1.14	11	0.90
	*HIS3 - kanMX*	224	0	79	26.1	13.0+/−0.13	14.5	0.90
	*MAT- kanMX*	167	2	139	46.4	24.5+/−1.9	25.1	0.98

PD-parental ditype, NPD-non parental ditype, TT-tetratype, ^1^cM-centimorgans, cM were calculated by the Perkins formula: cM = 50(T+6 NPD)/(T+NPD+PD) [62]; ^2^Only distances with homology present are shown. ^3^Insert is hemizygous. ^4^Insert has homology at *CAN1* ORF

When diploid cells were induced to undergo meiosis the mutation rate was 37×10^−8^ (Table 2, GRY3262), a 6.5-fold increase from the mitotic diploid rate (p = 2×10^−8^). Our data are in agreement with the early observations from Magni and Von Borstel [28]–[30], where they observed a 6–20 fold increase in mutation rates after the induction of meiosis. Therefore, we conclude that we see the meiotic effect in our system.

### The meiotic effect requires Spo11

To determine whether the increase in mutations that occurred during meiosis was a consequence of meiotic recombination, we constructed diploid strains that were homozygous for *spo13* or both *spo13* and *spo11*. Cells mutated in *spo11* are unable to sporulate, however, a concomitant mutation in *spo13*, which allows bypass of meiosis I, overcomes the sporulation defect of *spo11* mutants, resulting in two diploid spores [40]. Results of this analysis are shown in Table 2 (Strains GY3273, GRY3274 and GRY 3275). The mutation rates during mitotic growth are similar for all three strains, although ∼2 fold lower in the *spo11/spo11* diploid. Homozygous diploid *spo13/spo13* strains show a 5.8 -fold increase in the mutation rate after meiosis, similar to the increase in the wildtype strain. In our strain background viability was significantly reduced upon induction of sporulation in the *spo13Δ* diploids (to ∼10%), requiring an increase in the volume of our starting cultures. This inviability was rescued by a concomitant *spo11* mutation as had been previously observed [41]. The *spo11 spo13* diploids did not result in an increase in the mutation rate after induction of sporulation. These data provide strong support for the role of recombination and specifically, Spo11 meiotically induced DSBs, in the meiotic effect.

### Rev3 is responsible for half of the meiotically induced mutations

Because Polζ is one of the primary polymerases responsible for the majority of both spontaneous and induced mutations in yeast [24], and is up-regulated during meiosis [27], [42] we analyzed the role of Rev3 on the meiotic effect. The results are shown in Table 2 (Strain GRY3276). As expected, Rev3 appears to be responsible for one-half to two-thirds of the spontaneous mitotic events: in haploids there were about twice as many His^+^ Can^r^ events in the wild type strain (2.8×10^−8^ Table 2, GRY2691) as in the *rev3* strain (0.8×10^−8^, GRY3265). Similarly, there was a 3-fold difference in the mutation rates during mitotic growth in diploid strains from 5.7×10^−8^ in the wild type, versus 2.0×10^−8^ in the *rev3* strain.

Induction of meiosis still results in a large increase in the mutation rate. The increase in *rev3* strains was 8-fold higher after meiosis as compared to the mitotic mutation rate (Table 2, GRY3276, 2×10^−8^ versus 16×10^−8^). Because we observe no differences in the frequency of recombination, sporulation or viability of spores in the rev3 mutant strains, we do not think that Rev3 influences the frequency of Spo11 induced breaks, although this has not been directly tested. Assuming that the efficiency of breaks is not affected between wildtype and *rev3* mutants, we consider a better comparison is between the meiotic rates in the wild type strain (Table 2, 36×10^−8^, GRY3262) and the *rev3* strain (Table 2, 16×10^−8^, GRY3276). In this comparison the mutation rate in the *rev3* diploid is only about half the expected rate if Rev3 had no role in the meiotic effect. Thus, as in spontaneous mitotic mutations, Polζ appears to be responsible for introducing about half of the meiotic mutations. We saw little difference in recombination between the markers tested in the wild type and *rev3* strains by tetrad analysis (Table 3, Strain GRY3276, p>0.4). The *natMX-MAT* interval is 0.16 cM/Kb for the wild type versus 0.27 cM/kb for the *rev3* strain. This observation further supports that Rev3 does not influence the formation of meiotic DSBs per se, but is an important player in introducing mutations during the repair of the breaks when necessary.

### Sequence analysis of mutational events in mitosis and meiosis

We sequenced ∼80 independent *can1* mutants to determine whether there are any obvious mechanistic differences between mutations generated during mitosis or meiosis. A summary of the sequence analysis is shown in Table 4, and the data are shown in Supplementary S1 Table and S2 Table. There was very little noticeable difference between mutants generated during mitotic growth and those generated during meiosis. There were slightly more frame-shift mutations in meiosis as compared to mitosis (p = 0.02). There was no noticeable change in the distribution of mutations along the *CAN1* gene (p = 0.85).

**Table 4:** Summary of mutation spectra for mitotic and meiotic cell divisions^1^.

	Frameshift		Base substitution		Complex
	Frequency (%)	Type^2^	Frequency (%)	Type^3^	Frequency (%)
Mitosis	8/80 (10)	6∶2	72/80 (90)	41∶31	0/80 (<1.3)
Meiosis	20/96 (21)	15∶5	72/96 (75)	44∶31	4/96 (4)

1 Data are from strain GRY3262, Fig. 1C.

2 contractions∶expansions.

3 transitions∶transversions.

### Analysis of a reporter with *CAN1* ORF homology

One caveat to our experimental design is that the 3.8 kb cassette is hemizygous for *CAN1* and could potentially influence both the frequency and types of meiotic events. To determine if the presence of increased homology might influence meiotic recombination rates and mutagenesis in our system, we designed a related cassette to provide homology to the *CAN1* ORF on the homologous chromosome. This insertion includes the entire *can1* gene with the exception of the promoter and the first 6 codons and increases homology by 2.6 kb. *LEU2* is substituted for *HIS3* (Fig. 1D). Results from this construct are shown in Table 2 (GRY3263). Again, we found that haploids had a lower mitotic mutation rate than diploids (2.8×10^−8^ for GRY2691 versus 8.2×10^−8^ for GRY3263, Table 2). We analyzed 192 mitotic His^+^ Can^r^ events for crossovers as described in Materials and Methods, and found no events with a crossover in the *natMX-MAT* interval.

The increased homology resulted in more meiotic crossovers in the *natMX-HIS3* interval, consistent with the 2.6 kb more homology where crossovers can occur. In unselected tetrads 7/252 (2.8%) had crossovers in the hemizygous strain (Table 3, GRY3262), and 12/245 (4.9%) had crossovers in the strain with *can1* homology (Table 3, GRY3263; p = 0.02). However, the increase in length did not affect overall recombination in the *natMX-HIS3* interval (0.18 cM/kb for the hemizygous strain GRY3262 vs 0.24 cM/kb for the strain with *can1* homology GRY3263, Table 3). Likewise, there was no significant difference in crossover frequency between the two strains in the *HIS3-MAT* interval (p = 0.3).

The presence of homology to *CAN1* did not eliminate the meiotic effect. Induction of meiosis resulted in a 5.9 fold increase in the mutation rate (Table 2, GRY3263, 49×10^−8^) compared with a 6.5 fold increase in *can1* mutations after meiosis in the hemizygous strain (Table 2, GRY3262, 36×10^−8^). We conclude that the meiotic effect is independent of the presence of a homolog for the *CAN1* ORF. It is true that there remains heterozygosity between *HIS3* and *LEU2*, and it is possible it influences the types of events seen. However, for the vast majority of cells that have undergone meiosis, the presence of heterozygocity at *CAN1* seems to have no effect on the recombination frequency in the area near the insertion of the *CAN1 HIS3* cassette (see below).

### Increased mutations at a meiotic hotspot

Meiotic recombination varies widely along the chromosome, resulting in coldspots and hotspots that correlate with the level of Spo11 induced breaks [10]. We predicted that since Spo11 is required for meiotic recombination, the rate of mutation induction during meiosis would also be influenced by the relative frequency of Spo11 DSBs. To test this prediction we inserted the *CAN1 HIS3* reporter close to a known meiotic hotspot between the *BUD23* and *ARE1* genes [43] 11 kb distal to *MAT*a (Fig. 1E, GRY3625). The *LEU2 can1* cassette described in the last section, was inserted at the same location on the *MATα* chromosome to provide homology to the *CAN1* ORF (Fig. 1E-GRY3626). We used a *kanMX* knockout of *YIH1* from the knockout collection [44] as an 11.6 kb distal marker for monitoring crossovers.

The mutation rates for the strain with the *HIS3 CAN1* cassette located between *BUD23* and *ARE1* are shown in Table 2 (GRY3630). There was a 3-fold increase in the mutation rate in diploids (GRY3630, 8.4×10^−8^) as compared to haploids (GRY3625, 2.5×10^−8^). Only 1/183 His^+^ Can^r^ mitotic events from GRY3630 showed evidence of a crossover between *HIS3* and *yih1::kanMX*.

To ensure that the insertion of the reporter did not affect the levels of meiotic recombination at the hotspot, we dissected tetrads from the resulting diploid strain, and compared the frequency of crossovers in the *MAT-yih1*::*kanMX* interval to that of a strain lacking the insertion. In the absence of the reporter construct, the interval between *MAT* and *yih1::kanMX* was 26.3 cM (1.2 cM/Kb GRY3629, Table 3). Previous meiotic data indicated that the interval between *THR4* and *MAT* was 1.2 cM/kb (http://www.yeastgenome.org/cgi-bin/geneticData/displayTwoPoint?locus=S000029699). Since *THR4* is 7.5 kb closer to *MAT* than *YIH1*, it is likely that most of the recombination occurs in the vicinity of the *BUD23-ARE1* hotspot. When we inserted the *HIS3 CAN1* cassette near the hotspot, recombination between *MAT* and *yih1::kanMX* was 24.5 cM (Table 3, GRY3630) resulting in 0.98 cM/Kb. Thus, although the insertion did cause a reduction of recombination at the hotspot, recombination was still about twice as frequent as the average for chromosome III (0.48 cM/Kb), and three-fold more frequent than the coldspot insertion between *natMX* and *MAT* (0.33 cM/kb, GRY3263).

**Figure 2:**
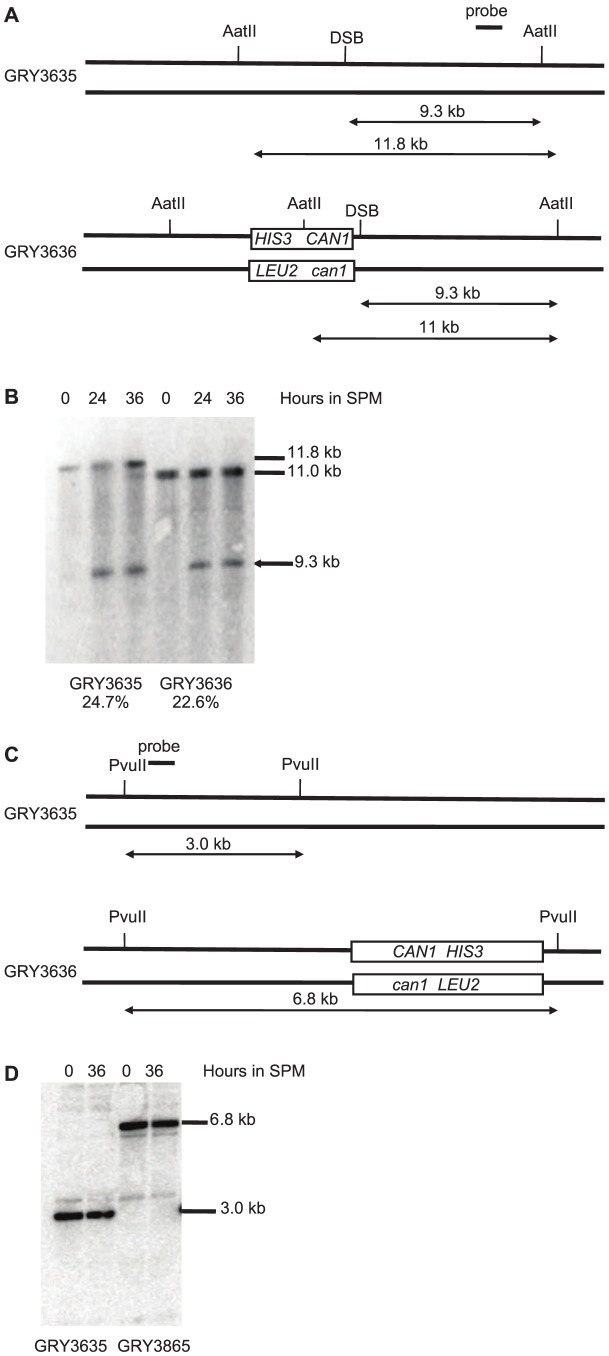
Southern blot analysis of meiotic hotspot DSB. **A.** Schematic of strains with and without the substrate showing restriction sites and probe locations. **B.** Meiosis time course of *sae2Δ* strains without and with the *HIS3 CAN1* cassette in the hotspot. Time (hours) after transfer to sporulation media (SPM). **C.** Schematic of strains with and without the substrate in the coldspot, showing restriction sites and probe locations. **D.** Meiosis time course of *sae2Δ* strains without and with the *HIS3 CAN1* cassette in the hotspot. Time (hours) after transfer to sporulation media (SPM).

Meiotic DSBs can be monitored and quantified in strains deficient for *sae2*, as these strains are unable to remove the bound Spo11 and initiate resection allowing the DSB to accumulate as unique bands [37], [45]. In strains lacking the reporter construct (Fig. 2A. GRY3635) 24.7% of the DNA accumulated a DSB in the *BUD23-ARE1* interval. When the reporter cassette is inserted nearby (Strain GRY3636), the level of DSBs was is 23.6%, consistent with similar levels of meiotic DSBs in the two strains (Fig. 2B). No breaks were detectable near the *HIS3 CAN1* cassette located in the coldspot (Fig. 2C). This is consistent with the observations of Pan et al [38] that they observed almost 10,000 Spo11 associated oligomers in the 6 kb surrounding the insertion site when it was in the hotspot, but only 384 in the 6 kb surrounding the insertion site at the coldspot.

The induction of sporulation resulted in a 3.6 fold increase in the mutation rate when the substrate was inserted in the hotspot (177×10^−8^, GRY3630, Table 2) versus when it was inserted in the coldspot (49×10^−8^, GRY3263, Table 2). This correlates well with the differences in meiotic recombination at the two loci (Table 3) either with or without the substrate. When the substrate was inserted near the coldspot (GRY3262) recombination was 0.33 kb/cM, versus 0.90 cM/kb when the substrate was inserted near the hotspot (GRY3630), a three-fold difference. Therefore, there is a positive correlation between meiotically induced DSBs and meiotically induced mutations.

### Meiotically induced mutations are more likely to be associated with a crossover

Although elevated, the frequency of meiotic mutation was too low to determine the crossover (CO) association by tetrad analysis. Therefore we examined red (*ade2-1*) cyh^r^ random spore colonies as described in Materials and Methods.

A direct comparison of crossovers between unselected tetrads (none of which had a mutation in *CAN1*) and the selected His^+^ Can^r^ random spores was complicated by the fact that from the random spores we cannot distinguish between a gene conversion (GC) event versus a double CO of a central marker(s), or a CO versus a GC of an outside marker. Therefore, we also examined random spores from canavanine sensitive (Can^s^) His^+^ spores.

**Figure 3:**
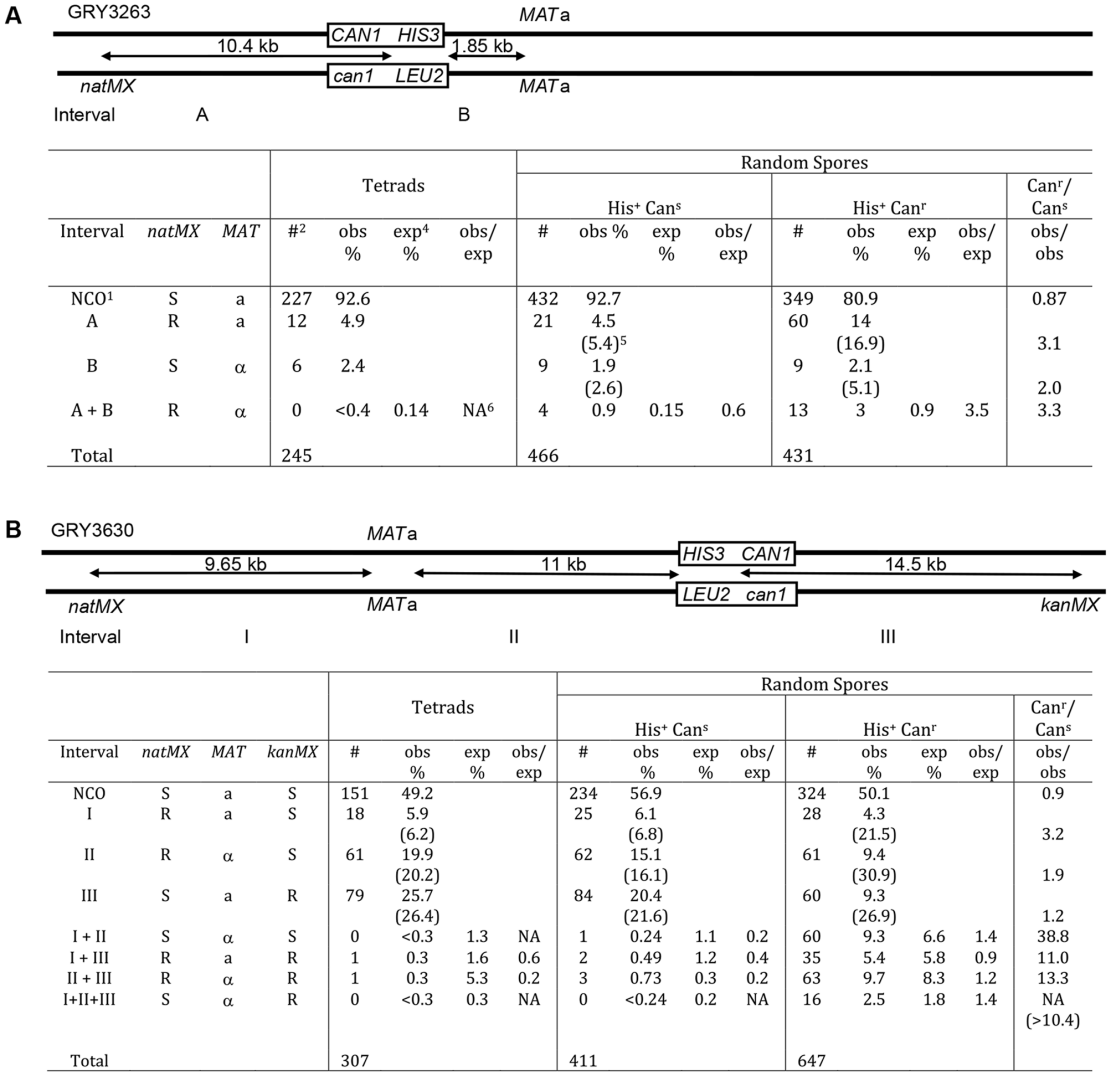
Crossover association among non-mutant (Can^s^) and mutant (Can^r^) spores. ^1^NCO: non crossover; ^2^#: number of events observed in each category; ^3^obs %: percent of total events observed (numbers in parentheses include double crossover events for the interval); ^4^exp%: percent of double crossover events expected based on genetic distance from observed total crossovers; ^5^NA: not applicable (since no events were observed). **A.** Spores from strains with the substrate in the coldspot were analyzed for crossovers in intervals “A” (*natMX–HIS3*), interval “B” (*HIS3-MAT*), and for double crossovers (crossovers in both interval A and interval B). Tetrads: data from tetrad analysis (Table 3), none of the spores from tetrad analysis had a mutation in *CAN1*. The expected double crossover frequency is based on the total number of observed crossovers in intervals A and B (numbers in parentheses). Random spores were classified as either His^+^Can^s^ (nonmutant) or His^+^Can^r^ (mutant). Can^r^/Can^s^ is a comparison of the observed crossovers among the His^+^Can^r^ random spores, divided by the observed crossovers among the His^+^Can^s^ random spores. There are significantly more crossovers among the mutant spores. **B.** Spores from strains with the substrate in the hotspot were analyzed for crossovers in intervals “I” (*natMX–MAT*), “II” (*MAT–HIS3*), “III” (*HIS3 – kanMX*) as well as double and triple crossovers.

A comparison of the data between tetrads and random spores for the strain with the substrate in the coldspot (GRY3263) is shown in Fig. 3A. The difference between recombination events in tetrads and His^+^ Can^s^ random spores was not significantly different (His^+^ Can^s^/Tetrads; p = 0.5). In contrast the spores that have had a mutation in *can1* (His^+^ Can^r^) were two-to three fold more likely to have had a crossover than either the tetrads, (Fig. 3A, p = 6.7×10^−5^) or the His^+^ Can^s^ random spores (Can^r^/Can^s^; p = 8.3×10^−7^). This difference was primarily due to an increase in events in the *natMX–HIS3* interval (A) and apparent double crossovers (A+B). The expected percent of double crossovers is calculated based on total recombinants with a crossover in an interval (numbers in parentheses). There are insufficient tetrads to determine whether there is any interference among the tetrads. However, there appears to be a loss of interference among the His^+^ Can^r^ random spores, although this could also be due to gene conversions that are counted as crossovers.

A similar analysis for the strain with the reporter cassette at the hotspot (GRY3630) is shown in Fig. 3B. The pattern of recombinants seen in His^+^ Can^s^ total random spores was not significantly different from the pattern of recombinants in tetrads (His^+^ Can^s^/tetrads; p = 0.25). However, there was a significant increase (10–40 fold) in crossovers among the His^+^ Can^r^ spores as compared to either tetrads or the His^+^ Can^s^ spores (Can^r^/Can^s^; p<1×10^−8^). Interestingly, the interval showing the greatest increase in crossovers is interval I. which is the furthest from the site of the break (>14 kb). The presence of interference is evident among the tetrads and the random spores, where the observed versus expected (obs/exp) ratio is <1. However there does not appear to be any interference among the His^+^ Can^r^ random spores where the obs/exp ratio is close to 1. We conclude that events that have acquired a mutation are ∼3 times more likely to be associated with a crossover than events that did not result in a mutation. Also, these events appear to be associated with crossovers that are quite distant from the initiating DSB.

## Discussion

Evolution is driven by the accumulation of mutations that are passed on in the germ line. Survival in evolutionary time scales involves providing sufficient variability so that adaptation can occur with a changing world. Most organisms ensure variability by maintaining a sexual lifestyle despite the cost. Here we explore the concept that the process of meiosis itself may be mutagenic and may also contribute to variability.

Support for this hypothesis was first documented in the early 1960′s by Magni and von Borstel [28]–[30] see Introduction). Here, we have revisited the meiotic effect with new tools and knowledge in hand, and have attempted to address the caveats present in previous work, as well as provide new data about the mechanism by which meiotic mutations arise.

In agreement with the observations of Magni et al [28]–[30], we find a 4 to 8 -fold increase in the *CAN1* mutation rate after the induction of meiosis. Several other groups have attempted to repeat the Magni observerations with little success. Whelan et al [46] also recognized the difficulty of measuring the appropriate diploid rate for *can1* mutations, and assumed that the diploid was either equal to the haploid, or twice the haploid rate. They did not subtract the frequency of the mutations generated during mitotic growth from each culture prior to determining the frequency/rate of mutations generated in meiosis. Whereas the events accumulate over several generations during mitosis, all of the meiotic events must accumulate in a single cell division, and therefore will be masked without this adjustment. In a high throughput sequencing approach, Nishant et al [47] found that the mutation rate during mitotic growth was similar to that previously determined. Because of the rarity of mutational events that occur during meiosis, they were only able to estimate that the global meiotic mutation rate in yeast was somewhere between zero to 55-fold higher than the mutation rate during mitotic growth (and thus well within any observed meiotic effect). Finally Qi et al [48] did deep sequencing of a cross between S288C and RMI11-1 (that diverge by 0.5–1%) and fully sequenced the products of one tetrad. They determined that the limit of their detection was ∼8×10^−8^/per base per cell division, and thus a 6–20-fold increase of the estimated global rate of mutations is still about 10-fold lower than they could detect.

The majority of the events that we sequenced were point mutations in the *CAN1* ORF and there appeared to be little difference between mutations made during mitosis or meiosis (see Supplementary Tables S1 and S2).

The presence or absence of homology at the *CAN1* ORF did not seem to affect the overall frequency of meiotic recombination, albeit it is impossible to determine whether the presence of any heterozygosity can influence the meiotic mutation rate. We see no significant change in sporulation and/or viability, nor in recombination due to the presence of our heterologies. Without an insert, we cannot measure the meiotic effect in our system. However, the fact that our observations are similar to those of Magni and von Borstel when they measure reversion of a recessive allele [28] suggests that the heterology itself is not inducing the meiotic effect.

By comparing the same substrates present in either the hotspot or coldspot, we find that when the substrate is near a coldspot, recombination is 0.24 cM/kb and the mutation rate induced by meiosis is increased ∼6 fold. When the substrate is located near a meiotic hotspot recombination is 0.9 cM/kb, and the mutation rate is increased 21 fold. Thus, there is a three- to four -fold increase in the level of recombination between the coldspot and the hotspot, and a three- to four-fold increase in the mutation rate. Associated crossovers are 2–3 times more likely to be found among the selected mutant spores than among non-mutants (Fig. 3A Can^r^/Can^s^) when the substrate is near the coldspot. When the substrate is in the hotspot crossovers are >10 fold more frequent among the mutant spores than non-mutant spores (Fig. 3B Can^r^/Can^s^). The largest increase in recombinants is in the interval furthest from the DSB (interval I), where 6.8% of the Can^s^ spores have a crossover and 21.5% of the Can^r^ spores have a crossover.

Meiotic crossovers show interference when the number of double crossovers is less than expected for an interval [12]. It is not clear how interference operates, but the evidence points to very early stages of recombination, possibly at the strand invasion step [49]. The influence of interference on crossovers is quite evident when looking at the strain with the substrate in the hotspot (GRY3630, Fig. 3B), where the observed/expected ratio for both tetrads and His^+^ Can^s^ random spores is below one.

In contrast, there appears to be a complete loss of interference among the His^+^ Can^r^ spores, where the observed/expected ratio is actually slightly >1. The dramatic increase (10–40 fold) in double and triple crossovers is particularly evident when one compares the % observed crossovers between the Can^s^ and Can^r^ mutant spores when the substrate is in the hotspot (Fig. 3B, Can^r^/Can^s^). In agreement with these observations is our key finding that the meiotic effect is dependent upon the presence of Spo11, the protein that introduces meiotic DSBs.

Because we cannot distinguish between gene conversion events or double crossovers among the random spores, one possibility is that the events that result in a mutation at *CAN1* are unusual in that they are associated with long resection. This is suggested by the fact that at the hotspot, interval I, >14 kb from the site of the DSB (Fig. 3B), has the highest increase in crossovers. Increased resection could have several consequences: a) increased ssDNA that is more susceptible to DNA damage, b) increased gene conversion tract lengths that might be confused with crossovers in our random spore analysis, c) template switching as has been seen in BIR or d) a loss of crossover interference. The average gene conversion tract in meiosis is ∼1.8–2 kb [50], well below the 14 kb distance of interval I from the break site.

We find that about one half of the mutations produced in meiosis are dependent upon Rev3, a component of the Polζ translesion DNA polymerase. This effect is not very different than that seen for spontaneous mitotic mutations. In contrast, DSB induced mitotic mutations vary significantly in their dependence upon Rev3, suggesting that context is of key importance for its activity (see Introduction). We saw no evidence for any effect on the frequency, viability, or meiotic recombination in a *rev3* mutant as compared with the wildtype, leading us to assume that Rev3 is unlikely to be affecting the rate of Spo11 breakage.

If the mutational events do result from longer regions of ssDNA, it is possible that this leads to increased damage, and therefore a potential direct role for Polζ in introducing some of the mutations during lesion bypass synthesis. Mutations occurring long distances (>8 kb) from the initiating lesion have been observed in other DSB associated assays [17], [18], [20], suggesting that rare mutational events are associated with exceptional events. The occurrence of multiple template switch events has also been documented during BIR, or when homology is limiting [51], [52]. These types of events would appear as double or triple crossovers in our random spore analysis. Clearly there is at minimum a loss of crossover interference. Longer resection tracts are most likely associated with delayed repair thereby potentially leading to an uncoupling from the mechanism of crossover interference. If longer resection tracts are associated with mutation, it is possible that a role for Polζ is in copying over DNA damage that might arise during the single stranded phase of repair. However, we cannot distinguish whether the drop in mutations after meiosis seen in the absence of Rev3 is due to repair by an error free mechanism, or whether the cells cannot traverse the lesion and die.

Most mutations are thought to be detrimental, and cells have gone to great lengths to keep mutations at a minimum by having multiple repair pathways to deal with the plethora of different lesions that they encounter. So what then is the point of allowing the mutational load to increase, albeit still at a very low level, during meiosis? We entertain three models. First, perhaps this is part of the compromise organisms make to help maintain variability in the population. The increased error rate may be an unavoidable consequence of the DSB pathway used to initiate meiotic exchange and the advantages of meiosis outweigh the added mutation load. Second, the option to increase mutagenesis during meiosis may have advantages in the sense of increasing the diversity of the germ cell pool. The increased mutational load allows for novel alleles to appear that might have selective advantages. A more provocative third model is that the meiotic effect allows organisms to direct the location of the genes subject to elevated mutagenesis. One of the oddities of meiotic recombination is that there are chromosomal hotspots and coldspots, and the position of these may be highly conserved [50], [53]. Thus organisms could increase the evolutionary rates of genes by controlling whether they were situated near hot spots of meiotic recombination or protect them from this process by preserving them in cold spots. Indeed, in a survey of yeast genes it was found that essential genes tended to be clustered with each other and are generally cold for meiotic recombination [54]. Since recombination is initiated by DNA double strand breaks, and breaks are usually the recipients of genetic information, it is a conundrum as to how hotspots are maintained. For example, a recent study of several isolated wild strains of *Saccharomyces paradoxus* indicates that the recombination hotspots are found at similar locations between the evolutionarily separated species *S*. *cerevisiae* [53]. It is worth noting that the generated data were exclusive to chromosome III and was obtained by PFGE, therefore at low resolution. A more recent global analysis of DSB sites in yeast suggests that hotspots are located in chromatin-depleted regions that are usually associated with some promoters and active genes [38]. Thus, it is probable that hotspots are conserved because of the underlying structural organization of the genome.

Since it appears that meiotic hotspots may be maintained on a global scale, this would allow cells to regulate the position of genes, or more likely the region of meiotic DSB sites so that essential genes are near coldspots, whereas genes where increased variability is desirable are near hot spots. In a high-resolution meiotic mapping experiment in a diploid of two *S. cerevisiae* haploid strains with 0.5% heterology, crossover sites were found to coincide with previously mapped DSB hotspots and with sites of increased variability among yeast strains [50]. On the other hand, Noor [55] found no evidence for increased genomic variability near hotspots between *S. cerevisiae* and *S. paradoxus* strains. It is important to note that his conclusions are based on assuming identical hot and cold spots between the two strains, despite the only data suggesting this comes from the low-resolution map of chromosome III by Tsai et al [53]. In contrast, there is an excellent correlation between recombination and sequence divergence in *Drosophila* [56]. In mammals recombination hotspots are associated with increased SNPs [57]. One major caveat is that recombination is easier to recognize when more SNPs are present. As the positions of meiotic hot spots are determined in more organisms it will be of interest to see whether the proposed correlation of hot spots with gene evolution rates is validated.

In summary, we have shown that mutations are increased during meiosis and that these results correlate with increased recombination events and are dependent upon the protein responsible for initiating meiotic recombination. We suggest that these are meiotic events that have gone awry, leading to increased resection, increased DNA damage, and loss of crossover interference.

## Materials and Methods

### Media

*S. cerevisiae* cells were grown in YEPD (Sherman et al. 1986) or the appropriate AA-synthetic drop-out media. AA drop-out media is similar to SD media described by Sherman et al. (1986) except that all amino acids, uracil, adenine, Myo-inisitol are 85 µg/mL, except for leucine, which is at 170 µg/mL, and para-aminobenzoic acid and which is at 17 µg/mL. Drop out plates were only missing the noted amino acid. Canavanine was added at 100 µg/ml and cyclohexamide at 5 µg/ml. To identify red colonies on minimal media the adenine was reduced to 20 µg/ml. Amar spore medium is 2% potassium acetate supplemented with 100 µg/ml adenine and uracil, 50 µg/ml histidine, leucine, lysine, tryptophan, methionine and arginine, 35 µg/ml phenylalanine and 10 µg/ml proline, and is designed to support sporulation without growth of the culture.

### Strains and strain construction

All strains used in this study are listed in Table 1. Strain GRY2691, a *MAT* a parent (Table 1) was constructed by transformation of GRY1600 with a PvuII fragment from plasmid pMush22 [14] containing *CAN1* and *HIS3* genes transcribing away from one another (Fig. 1A) resulting in an insertion of the 3.8 kb cassette 1.85 kb proximal to the *MAT* locus. The sequences of *CAN1* present in the cassette are from −148 to +1973 relative to the *CAN1* start codon. The strain also harbors a deletion of *CAN1* that excludes sequences from −151 to +1995 relative to the ATG, thus there is no homology present between the two loci. The *HIS3* insertion includes sequences from −191 to +857 of the *HIS3* ORF. The *his3-Δ200* mutation extends from −205 to +835, thus there are only 22 bp of homology on the 3′ end of *HIS3*, and no homology on the 5′ end. The *MATα* parent (GRY2690, Table 1, Fig. 1B) was constructed by insertion of a *natMX* cassette from pAG25 [58] between *FEN1* and *SNM1* on chromosome III and selection on nourseothricin (clonNAT, Werner BioAgents) essentially as described for creating the yeast knockout libraries by providing 45 bp of homology on either side of the cassette to the target locus [59]. The *kanMX* cassette was inserted into GRY2690 by PCR of *yih::kanMX* from the yeast *MAT*a knockout collection strain (Open Biosystems) with an additional ∼250 bp flanking homology and selection on G418 (Genticin, US Biologicals). To construct the promotorless *can1* gene (Fig. 1 C) we replaced *HIS3*, the *CAN1* promoter, and the first 6 amino acids of the *CAN1* ORF of pMush22 with a *LEU2* marker by recombineering [60] with a PCR fragment of *LEU2* containing 35 bp of flanking homology to either side of the *HIS3* gene. Once the construct was verified and sequenced, it was inserted into strain GRY2690 by one step transplacement [61]. Hotspot constructs were made by PCR of the *HIS3 CAN1* and the *LEU2 can1* cassettes with 5′ end- tailed primers containing 50 bp homology on either side to a site between *ARE1* and *BUD23*. The *ARE1* and *BUD23* genes are transcribed away from one another. The insertion did not delete any base pairs, and was positioned so that it was between −333 of *BUD23* and −147 of *ARE1*.

Strains deleted for *SPO11* and *SPO13* were constructed by PCR of the appropriate gene disruption from the yeast knockout collection [44] and subsequent transformation into strains GRY2690 and GRY2691 followed by selection on G418 as described [59]. Disruptions were confirmed by PCR, Southern blot and phenotypic analysis where possible. Strains deleted for *rev3* were obtained by one-step transplacement of an Xba1 fragment containing a *rev3::LEU2* disruption from plasmid pAM56 (kindly provided by Alan Morrison). Transformants were selected on media lacking leucine, and further confirmed by PCR and a reduced level of UV induced papillation to canavanine resistance for strains carrying the *HIS3-CAN1* cassette. Strains deleted for *sae2* were PCR amplified from a *sae2::*HygMX mutant strain, which was constructed by marker replacement of the knockout collection [44], [58].

### Tetrad analysis

Tetrads were isolated by patching the various diploid strains on YEPD, then growing up a 5 ml culture in Amar spore media for a minimum of 5 days. Tetrads were treated with zymolyase and dissected onto YEPD. After growth on YEPD, the tetrads were examined for each of the relevant markers by replica plating. Genetic distance was calculated using the Perkins equation (cM = 100X =  (100 (6N+T))/(2(P+N+T)) [62].

### Fluctuation tests and random spore analysis to determine the mutation frequency in mitotic and meiotic diploids

To analyze the rate of the mutations occurring during mitotic cell divisions we used a Luria-Delbruck fluctuation test [63] performed as follows: The relevant haploid strains were mated, and zygotes were isolated by micromanipulation on YEPD. Individual zygote colonies were struck for single cells on YEPD. 9 colonies from each independent zygote colony were inoculated into 5 ml of YEPD. Cells were shaken at 30° overnight and then diluted 1∶50 into 10 ml fresh YEPD and incubated for ∼4–6 hours at 30° to mid-log phase (2–4×10^7^ cells/ml) with shaking (for the *spo13* diploids, we grew 100 ml of culture). After washing cultures in sterile water, half of the cells were removed and used to determine the number of His^+^ cells and the number of His^+^ Can^r^ mutants from each culture. The remaining diploid cells were then resuspended into 5 ml sporulation media and incubated at 30° with shaking until >90% of the cells had sporulated by microscopic examination (∼5 days). Mutation rates during mitosis were estimated by the Ma-Sandri-Sarkar Maximum Likelihood Method [64]. This is a recursive algorithm that is the product of the probabilities *p_r_* for the experimental results *r* (the number of mutants per culture). *m* is the number of mutations per culture, and *c* is the number of cultures. The proportion of cultures with no mutations is 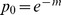, and cultures with 1,2…*i* mutations are calculated by 
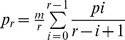
. The recursive function works as follows: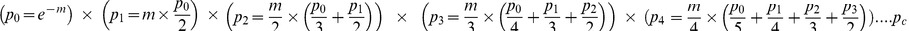 to find the best estimate of *m* from the fluctuation analysis. The mutation frequencies (His^+^ Can^r^/His^+^) ranged from ∼1×10^−5^ to 1×10^−8^ with majority of cultures falling in the 2–5×10^−7^ range. To determine the 95% confidence intervals we used the following equations 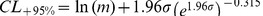 and 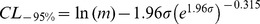 from Foster [65].

For determination of mutation rates during meiosis, we used a random spore analysis as described [66]. Briefly, The ascospores were washed twice in 5 ml water and then resuspended in 5 ml water with 0.25 ml 1 mg/ml Zymolyase-100T and 10 µl 2-ME. Cells were incubated overnight at 30°C with gentle shaking. 5 ml 1.5% NP40 (Roche # 11754599001) was added along with 2 ml acid washed glass beads and incubated on a roller drum at room temperature for 2 hours with occasional vigorous vortexing. Disruption of spores was monitored microscopically. This procedure lyses all unsporulated cells. Appropriate dilutions were plated onto media to determine the total number of His^+^ and His^+^ Can^r^ cells. Determination of the meiotic frequency/rate was calculated by subtracting the mitotic frequency from the meiotic frequency for each culture (Meiotic His^+^Can^r^/His^+^ per ml minus Mitotic His^+^Can^r^/His^+^ per ml). The resulting frequencies were then used to determine a median value reflecting the rate of mutations arising during meiosis, since meiosis involves a single division. For linkage analysis we selected doubly recessive red (*ade2-1*) Cyh^r^ His^+^ Can^r^ random spores to maximize analysis of haploid cells versus cells that mated after plating.

### Analysis and determination of crossovers among mitotic mutants

To identify crossovers among the mitotic mutants we patched ∼200 His^+^ Can^r^ events onto YPD, phenotypically scored the markers, and replica plated them to sporulation media. After 6–7 days at 30°, spore patches were replica plated to YPD, grown overnight and mated to freshly grown strains GRY633 and GRY634. Diploids were selected on media lacking both histidine and uracil. Because the *HIS3 CAN1* cassette is located near the *MAT*a locus, non-crossovers mate with strain GRY634 (*MATα*), and give rise to Nat^s^ colonies. Crossovers between *natMX* and *HIS3 can1* also only mate with strain GRY634 (*MATα*) but form Nat^r^ progeny. Finally, crossovers between *HIS3 can1* and *MAT* mate with strain GRY633 (*MAT*a) resulting in Nat^s^ colonies. Double crossovers between the same chromatids (of which none were detected) would mate with GRY633 and become Nat^r^. No crossovers between *HIS3* and *MAT* were observed among the mitotic diploids analyzed. A similar scheme was used to analyze for mitotic crossover events for the strains with the construct in the hotspot except that the *kanMX* marker was also scored.

### Analysis and determination of crossovers among meiotic mutants

After plating for random spores we colony purified 300–600 red His^+^ Can^r^ Cyh^r^ spore clones from each strain onto media lacking Histidine. These were then retested for Can^r^, patched and replica plated to test the relevant markers and determine mating type (by crossing with strains DC14 and DC17). Approximately 50 of the spore colonies from each strain that presented evidence of a crossover were mated to GRY1600 or GRY1601 (depending on mating type) and 5–10 tetrads were dissected from each to further confirm linkage.

### Sequence analysis of the *can1* mutants

The entire *CAN1* gene was PCR amplified from mutant candidates and sequenced on both strands. Three polymorphisms were noted in our *CAN1* gene as compared to the published S288c *CAN1* sequence. The sequence of the mutations identified in mitosis or meiosis are listed in Supplemental Tables S1 and S2, respectively. Sequencing was done by the Laboratory of Molecular Technologies Sequencing Facility- SAIC-Frederick, FNLCR.

### Southern blot analysis

DNA isolation and Southern blots were carried out as described by Sun et al. [67]. For the hotspot analysis DNA was digested with AatII and run on a 0.7% agarose gel. For the coldspot analysis DNA was digested with PvuII and run on a 1% agarose gel. ^32^P-dCTP labeled probes were made with the Agilent Prime it II kit according to manufacturers instructions. Hotspot probe used a PCR fragment spanning coordinates 213283–213848 of Chromosme III. Coldspot probes used a PCR fragment spanning 195735–196774 of Chromosome III.

### Statistical analysis

Statistical analyses were calculated by the chi square test for tetrads and random spores: in tetrads we compared PD, NPD and TT between the various strains, for random spore analysis we compared the number of crossovers in each interval for each strain. For distribution analysis along the length of *CAN1* the gene was divided into 200 bp windows as described previously [25]. For comparison between mutation rates from mitotic growth and from meiosis we used a student's *t* test (http://en.wikipedia.org/wiki/Student's_t-test).

## Supporting Information

S1 Table Sequence and location of *can1 HIS3* mutations generated during mitotic cell divisions.
(DOCX)

S2 Table Sequence and location of *can1 HIS3* mutations generated during meiotic divisions.
(DOCX)
