# Supplementary material for: Elevated Mutation Rate during Meiosis in Saccharomyces cerevisiae
Source: PLoS Genet. 2015 Jan 8;11(1):e1004910. doi: 10.1371/journal.pgen.1004910 (PMC4287439; doi:10.1371/journal.pgen.1004910)
Supplement: S1 Table — Sequence and location of can1 HIS3 mutations generated during mitotic cell divisions. (DOCX) [file pgen.1004910.s001.docx]

**Table S1- *can1* mutations generated at *HIS3 CAN1* during mitotic growth**

| Position relative to ATG of *CAN1* | DNA change | AA change |
| --- | --- | --- |
| 109 | 3G->4G | FS |
| 130 | G->T | E-> Stop |
| 217 | T-->C | No change |
| 222 | A->C | E->D |
| 223 | G->C | D->H |
| 238 | C->T | Q->Stop |
| 249 | 2A-->1A | FS |
| 268 | C->T | Q->Stop |
| 275 | A->C | H->P |
| 299 | G->A | G->A |
| 302 | C->A | T->I |
| 308 | G->T | G->V |
| 311 | C->G | T->R |
| 313 | T->G | G->C |
| 373 | T->C | S->P |
| 386 | T->G | M->R |
| 413 | A->T | Q->L |
| 424 | G->A | E->K |
| 430 | G->C | A->P |
| 452 | C->T | S->F |
| 472 | C->T | Q->Stop |
| 497 | G->A | G->D |
| 518 | T-->C | Y->H |
| 522 | G->A | W->Stop |
| 529 | T->C | W->R |
| 531 | G->T | W->C |
| 532 | G->C | No change |
| 544 | delG | FS |
| 553 | C->T | L->F |
| 626 | 6T->5T | FS |
| 659 | T-->C | V->A |
| 682 | G->C | E->D |
| 687 | G->A | W->Stop |
| 691 | G->A | A->T |
| 718 | G->A | G->R |
| 733 | G->T | No change |
| 734 | G->T | C->F |
| 740 | G->A | C->Y |
| 745 | delG | FS |
| 750 | T->A | C->Stop |
| 772 | G->C | V->L |
| 824 | delCT | FS |
| 858 | G->A | W->Stop |
| 892 | C->T | Q->Stop |
| 911 | G->A | G->D |
| 928 | G->A | A->S |
| 929 | C->G | A->G |
| 929 | C->G | A->G |
| 952 | C->T | P->S |
| 955 | A->T | R->Stop |
| 977 | T->C | F->S |
| 992 | T->C | F->S |
| 1011 | T-->G | L->Stop |
| 1026 | A->C | L->F |
| 1035 | C->A | Y->Stop |
| 1045 | A->T | K->Stop |
| 1130 | T->A | I->N |
| 1162 | T->C | S->P |
| 1173 | T->G | N->K |
| 1184 | A->G | Y->F |
| 1193 | C->G | S->C |
| 1212 | T->C | L->P |
| 1214 | C->G | S->Stpp |
| 1214 | C->T | S->L |
| 1224 | delG | fs |
| 1262 | G->A | G->D |
| 1388 | C->T | A->V |
| 1390 | T->C | W->R |
| 1392 | G->A | W->Stop |
| 1393 | G->A | W->Stop |
| 1481 | T->G | L->Stop |
| 1485 | G->T | M->I |
| 1487 | C->T | P->L |
| 1623 | G->A | W->Stop |
| 1661 | G->A | W->Stop |
| 1662 | G->A | W->Stop |
| 1703 | T->C | T->I |
