# Supplementary material for: Elevated Mutation Rate during Meiosis in Saccharomyces cerevisiae
Source: PLoS Genet. 2015 Jan 8;11(1):e1004910. doi: 10.1371/journal.pgen.1004910 (PMC4287439; doi:10.1371/journal.pgen.1004910)
Supplement: S2 Table — Sequence and location of can1 HIS3 mutations generated during meiotic divisions. (DOCX) [file pgen.1004910.s002.docx]

**Table S2 – *can1* mutations generated at *HIS3 CAN1* during meiosis**

| Position relative to ATG of *CAN1* | DNA Mutation | AA change |
| --- | --- | --- |
| 27 | AA->GG | QR->QG |
| 90 | C->T | No change |
| 97 | A->C | T->P |
| 109 | 3G->2G | FS |
| 137.3 | T->A | V->D |
| 142 | G->T | E->Stop |
| 155 | delG | FS |
| 238 | C->T | Q->Stop |
| 257 | G->A | R->K |
| 287 | T->C | I->T |
| 311 | C->A | T->I |
| 314 | G->A | G->D |
| 317 | 4T->5T | FS |
| 323 | A->T | H->L |
| 328 | repeat 18bp | TGLFIG |
| 352 | 2G->G | FS |
| 374 | C->A | S->Stop |
| 388 | G->A | V->I |
| 397 | G->C | A->P |
| 415 | T->C | S->P |
| 442 | G->A | E->K |
| 443 | C->T | P->L |
| 448 | C->A | H->N |
| 452 | C->T | S->F |
| 473 | C->T | T->I |
| 526 | T->C | S->P |
| 527 | C->A | S->Stop |
| 530 | G->T | W->L |
| 531 | G->C | W->G |
| 550 | G->A | E->K |
| 577 | C->T | Q->Stop |
| 584 | G->A | W->Stop |
| 611 | G->A | W->Stop |
| 612 | G->A | W->Stop |
| 625 | 6T->5T | FS |
| 626 | 6T->8T | FS |
| 657 | C->G | P->A |
| 661 | delAA | fs |
| 667 | T->C | Y->S |
| 669 | G->A | T->K |
| 670 | G->A | G->S |
| 680 | A->G | E->G |
| 690 | A->G | K->E |
| 691 | G->C | A->P |
| 709 | G->C | A->P |
| 718 | G->A | G->R |
| 733 | T->C | C->R |
| 736 | G->A | G->R |
| 740 | insC | FS |
| 762 | 4G->3G | FS |
| 785 | delT | fs |
| 805 | insC | FS |
| 807 | T->C | W->R |
| 808 | G->A | W->Stop |
| 812 | 4G->5G | FS |
| 858 | G->A | W->Stop |
| 890 | C->A | T->K |
| 891 | C->A | F->Y |
| 915 | G->C | G->R |
| 927 | G->A | G->S |
| 979 | C->G | R->G |
| 986 | T->A | L-> Stop |
| 995 | A->C | Y->S |
| 1020 | T->C | L->P |
| 1054 | C->T | Q->Stop |
| 1121 | T->A | L-> Stop |
| 1163 | C->G | S->C |
| 1173 | T->G | N->K |
| 1174 | T->C | S->P |
| 1175 | T->C | S->P |
| 1176 | C->T | S->L |
| 1183 | T->C | Y->H |
| 1184 | A->C | Y->S |
| 1193 | C->T | S->F |
| 1195 | C->T | R->C |
| 1214 | C->T | S->L |
| 1242 | delG | FS |
| 1244 | C->A | S->Stop |
| 1255 | A->T | K->Stop |
| 1262 | G->T | G->V |
| 1301 | G->A | G->D |
| 1310 | C->T | A->V |
| 1373 | T->G | V->G |
| 1374 | G->C | G->R |
| 1379 | G->C | G->A |
| 1381 | 5T->6T | fs |
| 1623 | G->A | W->Stop |
| 1689 | delG | fs |
| 1761 | T->G | N->K |
| 1762 | insA | FS |
| 284-301 | 17BP DEL | DEL/FS |
| 355-360 | CCAGTG -> TGAGCC | PRO->Stop  V->A |
